# Perceptions and experiences of medical student first responders: a mixed methods study

**DOI:** 10.1186/s12909-022-03791-z

**Published:** 2022-10-14

**Authors:** Andrew Orsi, Adam Watson, Nimali Wijegoonewardene, Vanessa Botan, Dylan Lloyd, Nic Dunbar, Zahid Asghar, A Niroshan Siriwardena

**Affiliations:** 1 Community and Health Research Unit, University of Lincoln, Lincoln, UK; 2 East Midlands School of Anaesthesia, Health Education England, Leeds, UK; 3 Medical Science Division, University of Oxford, Oxford, UK; 4 Directorate of Healthcare Quality and Safety, Ministry of Health, Colombo, Sri Lanka; 5 Medical School, University of Buckingham, Buckingham, UK; 6 South Central Ambulance Service NHS Foundation Trust, Otterbourne, UK

**Keywords:** Medical student, First responder, Medical emergency, Education, Training

## Abstract

**Context:**

Medical Student First Responders (MSFRs) are volunteers who respond to emergency calls, managing patients before ambulance staff attend. The MSFR role provides opportunities to manage acutely unwell patients in the prehospital environment, not usually offered as part of formal undergraduate medical education. There are few previous studies describing activities or experiences of MSFRs or exploring the potential educational benefits. We aimed to investigate the activity of MSFRs and explore their experiences, particularly from an educational perspective.

**Methods:**

We used a mixed methods design, combining quantitative analysis of ambulance dispatch data with qualitative semi-structured interviews of MSFRs. Dispatch data were from South Central and East Midlands Ambulance Service NHS Trusts from 1st January to 31st December 2019. Using propensity score matching, we compared incidents attended by MSFRs with those attended by other Community First Responders (CFRs) and ambulance staff. We interviewed MSFRs from five English (UK) medical schools in those regions about their experiences and perceptions and undertook thematic analysis supported by NVivo 12.

**Results:**

We included 1,939 patients (median age 58.0 years, 51% female) attended by MSFRs. Incidents attended were more urgent category calls (category 1 n = 299, 14.9% and category 2 n = 1,504, 77.6%), most commonly for chest pain (n = 275, 14.2%) and shortness of breath (n = 273, 14.1%). MSFRs were less likely to attend patients of white ethnicity compared to CFRs and ambulance staff, and more likely to attend incidents in areas of higher socioeconomic deprivation (IMD – index of multiple deprivation) (p < 0.05). Interviewees (n = 16) consistently described positive experiences which improved their clinical and communication skills.

**Conclusion:**

MSFRs’ attendance at serious medical emergencies provide a range of reported educational experiences and benefits. Further studies are needed to explore whether MSFR work confers demonstrable improvements in educational or clinical performance.

## Introduction

There is evidence that medical students and junior doctors feel underprepared to assess and manage acutely unwell patients and common medical emergencies [1–3]. Innovations to address this have included strengthening emergency medicine and prehospital care teaching in the undergraduate curriculum [4, 5], introducing mandatory emergency medicine clerkships [6] and other educational innovations such as prehospital care courses, [7] Emergency Medical Technician and scenario training [8], or programmes where students observe ambulance staff without participating in clinical care [9].

Another innovation, introduced in the last decade, is Medical Student First Responder (MSFR) schemes, through which medical students are trained to respond to emergency calls, independently manage patients prior to the arrival of an ambulance and to assist ambulance staff as part of a wider prehospital team [10, 11]. In the United Kingdom (UK), MSFRs provide a similar service to Community First Responders (CFRs), [12] who are uniformed lay volunteers that are trained, equipped and managed by ambulance services. Like CFRs, MSFR training is primarily focused on Basic Life Support (BLS), first aid and providing assistance to ambulance staff. In comparison to many international first responder systems, MSFRs and CFRs may use marked response vehicles and are dispatched to a wide variety of high priority medical emergencies to which ambulance staff are also sent [12].

MSFRs have operated since at least 2013 in England, when one well-described scheme organised by South Central Ambulance Service for medical students at the University of Oxford was established [10]. Since then, the number of schemes has grown, with at least 12 UK medical schools reportedly having volunteer MSFR schemes. Volunteering as an MSFR offers medical students the opportunity to manage acutely unwell patients in the prehospital environment, which they may not receive as part of their formal undergraduate medical education.

There is little evidence describing the activity or experiences of MSFRs and limited understanding of the educational benefits of MSFR work. The results of a previous survey of graduating MSFRs suggested that volunteering in this role might help medical students develop communication skills and the ability to manage medical emergencies [13]. Furthermore, medical students participating in prehospital care programmes also report increased acute care knowledge, interpersonal skills and interprofessional understanding [13]. MSFR schemes provide opportunities for learning and experience in acute care, so they may confer similar education benefits.

We aimed to investigate the activity of MSFRs and explore their experience of working in the prehospital environment to inform the future organisation of MSFR schemes and explore the educational opportunities for medical students participating in these schemes.

## Methods

### Research questions, design and setting

Our research questions were: What are the demographic and clinical characteristics of patients attended by MSFRs? What are the experiences of MSFRs joining an MSFR scheme, working as an MSFR and how does it affect their education, training and future role? We used a mixed methods explanatory sequential design [14], undertaking cross-sectional quantitative analysis of ambulance dispatch data to investigate MSFR activity together with qualitative semi-structured interviews of MSFRs to explore their experiences and perceptions of work. We collected routinely recorded dispatch data and electronic patient records from South Central Ambulance Service NHS Foundation Trust (SCAS) and East Midlands Ambulance Service NHS Trust (EMAS) for all incidents between 1st January 2019 and 31st December 2019. Using propensity score matching, we compared incidents and patients attended by MSFRs with those attended by CFRs and ambulance staff only (i.e. without CFRs or MSFRs attending). We then sought to understand and develop our quantitative findings with thematic analysis of interview data from MSFRs active across these two ambulance regions.

### Theory

We adopted pragmatism as our theoretical approach, combining and integrating quantitative and qualitative data, primarily out of a desire to produce research that is actionable and based on the activities and experiences of the MSFRs we were researching [15].

### Quantitative data collection

We collected anonymised data from the Advanced Medical Priority Dispatch System (used by EMAS), NHS Pathways (used by SCAS) and from electronic clinical records completed by ambulance staff from both services who attended incidents. Incidents where MSFR and CFRs attended were identified using resource call signs. Data variables for demographic and clinical characteristics available from incident patient records included date and time, response category, resource type dispatched (MSFR, CFR or ambulance staff only), chief complaint, incident postcode, patient demographics (age, sex, ethnicity), and whether patients were conveyed to hospital. In the UK, emergency calls are categorised according to clinical urgency from Category 1 (immediately life threatening, i.e. cardiac arrest), Category 2 (emergency, e.g. stroke or myocardial infarction), Category 3 (urgent, e.g. abdominal pain) to Category 4 or 5 (less urgent, e.g. diarrhoea and vomiting or back pain). We grouped patients into five age categories (≤ 19, 20–39, 40–59, 60–79, and ≥ 80 years), and categorised chief complaints as either cardiorespiratory, gastrointestinal / urinary, neurological / endocrine, injury / trauma, psychosocial / palliative or other. Incident postcodes were used to calculate an associated Index of Multiple Deprivation (IMD) decile where 1 represents the most socioeconomically deprived areas and 10 the least.

### Statistical analysis

Initial descriptive analysis showed that MSFRs most commonly responded in urban areas during the evenings; fewer than 2% of MSFR attendances were in rural areas compared with more than 20% of both CFR and ambulance staff attendances. We used propensity score matching to adjust for this imbalance, with propensity scores generated by regressing treatment on time of call and rurality [16]. One-to-one matching with a fixed calliper was performed to create pairs of MSFRs, CFRs and ambulance staff [16, 17]. The newly created samples had the same percentage of attendances in urban vs. rural areas and the same median time of call. After the propensity score matching, descriptive statistical analyses were used to present numbers and percentages of response categories, patient demographics and chief complaints attended by MSFRs, CFRs and ambulance staff only in order to describe the activity of MSFRs and compare this with the activity of CFRs and ambulance clinicians, using proportion tests to directly compare differences between these different resource types.

### Semi-structured interviews

We invited all current MSFRs and recently graduated former MSFRs volunteering with either EMAS or SCAS to be interviewed. We contacted these MSFRs through their MSFR scheme leadership team and also via advertisements on social media. All MSFRs who expressed an interest were interviewed. We aimed for an even split of interviews across EMAS and SCAS with a minimum target of 15 interviews. AW (a current MSFR), AO (a former MSFR) and NW (a medically qualified research fellow) individually interviewed MSFRs using a predesigned interview schedule. The schedule prompted discussion on key areas of interest, whilst encouraging participants to speak freely about their experiences. We conducted interviews by telephone or video-call in 2021. Interviews lasted for up to 45 min, were digitally recorded, and then transcribed verbatim.

### Thematic analysis

Thematic analysis was undertaken using the method of Braun and Clarke supported by NVivo 12 [18]. Interview transcripts were read, re-read and coded line by line. Two researchers (AW, NW) independently coded all transcripts, and these codes were then merged by a third researcher (AS). Codes were then grouped into themes and categories through a series of discussions involving the whole team. Final categories were decided through group consensus [19].

### Ethics

Ethical approval was obtained from the NHS Research Ethics Committee for data analysis and from the University of Lincoln ethics committee for interviews.

## Results

### Descriptive analysis of initial sample (before propensity score matching)

Data were analysed for 1939 patients attended by MSFRs, 22,495 patients attended by CFRs and 1,017,276 patients attended by ambulance staff. Initial analysis showed that MSFR attendances were overwhelmingly in urban areas (n = 1903, 98%) which was a far higher proportion than for ambulance staff (80%) or CFRs (68%). The median time spent on the scene of emergency calls for MSFRs was longer at 19:09 min for MSFRs compared to CFRs (15:48 min) or ambulance staff (15:42 min). Attending primarily urban calls much later in the day than CFRs and ambulance staff was a unique aspect of the medical student response in the initial data set that we analysed. The percentage of MSFRs arriving first on scene in the entire sample was 63.4% and the median time spent on scene was 30.40 min.

### Propensity score matched analysis

#### Patient characteristics

Patients attended by MSFRs (median age 58.0 years, 51% female) included 1,303 patients attended on behalf of EMAS (67.2%) and 636 on behalf of SCAS (32.8%). These were compared with equally sized samples for incidents attended by CFRs and ambulance staff adjusted for location and time imbalance using propensity score matching (Table 1). MSFRs were less likely to attend patients of white ethnicity compared to CFRs and ambulance staff alone, and more likely to attend incidents in areas with a high IMD (p < 0.05). There were non-significant trends towards MSFRs being more likely to attend patients aged 20–39 years and less likely to attend patients aged over 80 years.

**Table 1:** Patient characteristics of calls attended by MSFRs compared with CFR or ambulance staff only adjusted for rurality and call time

	MSFRs		CFRs (adjusted)		Ambulance staff (adjusted)	
	**n**	**%**	**n**	**%**	**n**	**%**
**Age group** (years)						
19 and below	202	12.04	195	11.60	184	12.29
20–39	417	24.85*	264	15.70*	300	20.04
40–59	385	22.94	337	20.05	295	19.71
60–79	361	21.51	437	26.00	363	24.25
80 and above	313	18.65*	448	26.65*	355	23.71
**Gender**						
Male	840	48.95	840	49.35	743	49.01
Female	876	51.05	862	50.65	773	50.99
**Ethnicity**						
Ethnic minority	147	13.61^(*)^	47	4.06^(*)^	81	8.35
Mixed	38	3.52	14	1.21	18	1.86
White	895	82.87**	1096	94.73**	871	89.79**
**Deprivation**						
High (IMD < = 5)	1370	70.65**	1047	54.00**	1175	60.60**
Low (IMD > 5)	569	29.35**	892	46.00**	764	39.40**

** p < 0.001, *p < 0.05, ^(*)^p = 0.07.

#### Response category

MSFRs primarily attended more urgent Category 1 (n = 299, 14.9%) and Category 2 (n = 1,504, 77.6%) incidents, with Category 2 incidents a greater proportion and Category 3 incidents a lesser proportion of their workload compared to ambulance staff (Table 2). The median MSFR response time for SCAS was 7.98 min (IQR [5.36, 12.00]) and 9.33 min (IQR [5.80, 13.96]) for EMAS.

#### Chief complaint

MSFRs attended a higher proportion of patients with cardiorespiratory or neurological/endocrine presentations in comparison with ambulance staff (Table 2). The two most common chief complaints attended by MSFRs were chest pain (n = 275, 14.2%) and shortness of breath (n = 273, 14.1%). MSFRs also frequently attended life-threatening emergencies such as unconscious patients (n = 157, 8.10%), seizures (n = 105, 5.42%), strokes (n = 52, 2.68%) and cardiac arrests (n = 50, 2.58%).

**Table 2:** Call categories and presenting complaints of patients attended by MSFRs compared with those attended by CFRs or ambulance staff only adjusted for rurality and call time

	MSFRs		CFRs (adjusted)		Ambulance staff (adjusted)	
	**n**	**%**	**n**	**%**	**n**	**%**
**Call Category**						
Category 1	288	14.86(*)	205	10.58	155	8.43(*)
Category 2	1504	77.61 **	1548	79.92	1074	58.40**
Category 3	129	6.66**	165	8.52	524	28.49**
Category 4	14	0.72	11	0.57	83	4.51
Category 5	3	0.15	8	0.41	3	0.16
**Chief Complaint Category**						
Cardio / Respiratory	720	37.50**	677	35.22	341	17.98**
Gastrointestinal / Urinary	53	2.76	76	3.95	106	5.59
Injury / Trauma	155	8.07	202	10.51	235	12.39
Neurological / Endocrine	331	17.24^(*)^	322	16.75	207	10.91^(*)^
Psychosocial / Palliative	59	3.07	70	3.64	97	5.11
Other	639	31.35**	575	29.91	911	48.02**

** p < 0.001 *p < 0.05 ^(*)^p = 0.06.

#### Qualitative interviews

**Fig. 1:**
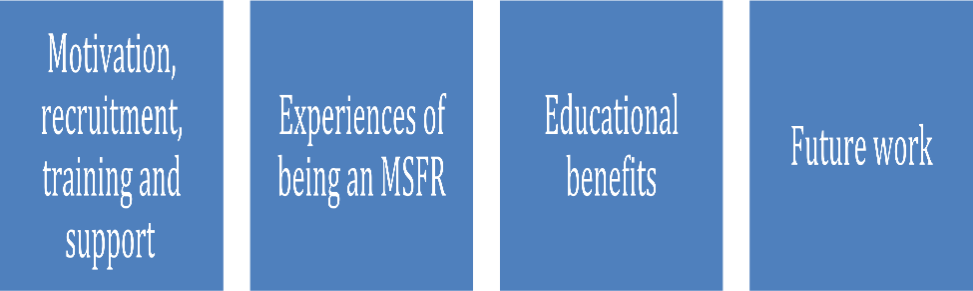
Thematic map summarising key themes arising from MSFR interviews

We interviewed 16 current and former MSFRs from five medical schools (Table 3). There are no published data on the total number of current or former MSFRs, but we estimate there are about 40 active MSFRs per medical school with around 200 active currently across the five medical schools included in this study. Our analytical themes were grouped into the following categories: motivation, recruitment and training and support, experience of being a MSFR, educational benefits, and future work (Fig. 1).

**Table 3:** Characteristics of MSFRs interviewed

Characteristic	n (%)
**Gender**	
Male	12 (75.0)
Female	4 (25.0)
**University**	
University of Nottingham (EMAS)	7 (43.8)
University of Leicester (EMAS)	1 (6.3)
University of Oxford (SCAS)	2 (12.5)
University of Buckingham (SCAS)	2 (12.5)
University of Southampton (SCAS)	4 (25.0)
**Medical School Year**	
2nd Year	4 (25.0)
3rd Year	1 (6.3)
4th Year	8 (50.0)
5th Year	2 (12.5)
Post-Graduation	1 (6.3)

### Motivation, recruitment, training and support

MSFR participants commonly identified two key motivations for applying to become an MSFR: an interest in a future career involving emergency medicine or prehospital emergency medicine and a desire to gain experience in managing acutely unwell patients before working as a doctor.

*“I was a bit scared of sick people in the sense that I was worried that I was going to become a doctor and not really know what to do when someone was deteriorating in front of me. I thought it would be a really good opportunity to actually get some practice.”* [Southampton MSFR, male]

MSFRs consistently described a competitive recruitment process involving combinations of application forms and interviews. Training varied in length and content between schemes, with a focus on Basic Life Support (BLS) and life-saving interventions such as use of an Automated External Defibrillator (AED). MSFRs generally agreed that additional training beyond the medical school curriculum was required for the role.

*“Training was very much geared towards an unresponsive patient”* [Nottingham MSFR, female]

We noted the importance of mentorship systems (where new MSFRs were paired with experienced responders), particularly in developing confidence. MSFRs also described regular recertification, peer-lead scenario training and development shifts with ambulance staff.

*“MSFRs who’ve already been in the scheme for a year or more can act as mentors and will take students out for their first few shifts”* [Oxford MSFR, male]

### Experience of being a Medical Student First Responder

MSFRs consistently reported highly positive experiences, with many describing the rewarding nature of the work and the contrast with typical clinical placements as medical students.

*“It’s also very difficult to finish a CFR shift and feel like a failure or that you’re useless or you haven’t contributed anything, because you have. You’ve gone and helped people when they needed it most.”* [Nottingham MSFR, female]

*“I don’t feel like it’s volunteering. I feel like it’s a clinical placement, but it’s also a clinical placement I enjoy and want to get up for, and I enjoy doing the shifts.”* [Southampton MSFR, male]

We also identified negative experiences reported by some MSFRs: competing demands on time, variability in MSFR workload and the perception of personal risk associated with responding, particularly at night or when working alone. Several MSFRs commented that the initial experience of new MSFRs was an important predictor of long-term commitment.

*“I think the most common reason for dropping out is not being sent to patients. So, recently there was someone who dropped out because they did three shifts and didn’t get dispatched to a single patient, so they gave up in the end.”* [Southampton MSFR, male]

*“I’ve definitely felt unsafe before, mostly when a member of the public phones, someone’s semi-conscious on the street, and you go and wake them up and they’re quite angry at you.*” [Nottingham MSFR, male]

### Educational benefits

MSFRs described numerous education benefits from their experiences. A common theme was a significantly increased exposure to acutely unwell patients, which MSFRs reported developed their competence and confidence in managing emergency situations.

*“You just get a lot more exposure. I find myself comparing it to what I’m doing at placement most of the time and the exposure to medical emergencies is just immense compared to what you do on placement.”* [Nottingham MSFR, male]

*“You’re always protected as a medical student, right? As a first responder, you can be going to almost anything.”* [Oxford MSFR, male]

We also noted that MSFRs credited their experiences with developing their clinicals skills, particularly in emergency situations they would not ordinarily encounter as medical students. In addition, MSFRs frequently commented on how the work had developed their communication with patients and other healthcare staff.

*“I’ve been to some really sick people. I’ve been to quite a lot of things like cardiac arrests where I’ve been the first person there on my own for 20 minutes.”* [Nottingham MSFR, male]

“*[Dr X], who used to run the medical school, said in the OSCEs, they could always see which students were doing CFR and which weren’t, because the practical skills were more advanced compared to the students that weren’t doing it.*” [Buckingham MSFR, female]

MSFRs also described a close working relation with local ambulance staff, with frequent informal teaching, and reported developing a better understanding of how pre-hospital care is delivered.

“*It’s also really nice that the paramedics and techs always want to teach you, they recognise that [we] are students, and that we’re still learning.*” [Nottingham MSFR, female]

*“I think, if nothing else, it’s helped me to see the bigger picture of medicine and see the whole journey the patient will have, as well as seeing what the ambulance service does in the community.”* [Leicester MSFR, male]

### Future work

MSFRs had mixed views regarding their scope of practice, with a number suggesting additional skills would be useful whilst others felt that a focus on basic interventions was most valuable.

*“I know that there are initiatives to equip some CFRs with ECG machines [...] That would be a good kind of jump.”* [Southampton MSFR, male]

“*You don’t want to be placing a huge amount of responsibility on a medical student to perform lots of interventions by themselves. That’s not the point of being a CFR. The point of being a CFR is that you do the basics well.*” [Nottingham MSFR, female]

A number of MSFRs described, how once qualified, they would have to cease MSFR work for medicolegal reasons related to areas such as licensing restrictions, insurance and supervision. These MSFRs went on to suggest that junior doctors might be able to provide a prehospital service like existing MSFR schemes if the appropriate medico-legal framework could be put in place.

*“We have to stop doing it when we graduate, and then have to stop for so many years before we can start doing it again in the capacity of a doctor. [...] I can’t see why, with maybe some additional training, [junior] doctors can’t do some of the interventions that, for example, maybe a paramedic or a registered pre-hospital clinician can do.”* [Nottingham MSFR, male]

## Discussion

### Main findings

This is the first study to use a mixed methods approach to describe the activities and experiences of MSFRs. Our data characterise the type of incidents that MSFRs commonly encounter and shows the wide scope and nature of their workload. As expected, MSFRs responded mainly to the most urgent categories of incident, with the most common presenting complaints being patients with chest pain or shortness of breath. MSFRs frequently attended serious life-threatening medical emergencies such as out-of-hospital cardiac arrests. These findings were expected as this reflected the dispatch criteria used by ambulance services when deciding whether to allocate MSFRs. During interviews with MSFRs, the opportunity to treat critically unwell patients was highlighted as an important motivator for being an MSFR, and also an important learning experience. MSFRs described how their responding had improved their clinical skills and confidence in managing medical emergencies. The variety of presenting clinical complaints and the frequency of high acuity calls were perceived to contribute to these benefits.

We found that MSFRs, compared with CFRs or ambulance staff attending alone, were more likely to attend patients from ethnic minority groups or living in areas with higher levels of deprivation. MSFRs were also more likely to attend younger patients (20–39 years old). In our interviews MSFRs reported mainly working in urban areas, close to their universities and student accommodation, so it is possible that the demographic differences seen in patients attended by MSFRs reflect the areas in which these medical students live and study.

### Comparison with previous literature

The motivation to work as an MSFR frequently appeared to revolve around either a pre-existing interest in emergency medicine or a desire to improve clinical skills [6]. Although strategies of integrating emergency medicine into the curriculum [20] such as providing education, [7] skills training [8, 21] and opportunities for observation 9 may be helpful to medical students, the MSFR role can further benefit students by providing first-hand experience of emergencies in the community while also benefitting patients by providing an additional emergency response [10]. Practising as an MSFR can address important gaps in procedural skills, such as basic life support [22].

MSFRs often learnt of the existence of schemes from other students, which suggests there is a perception that involvement in MSFR schemes benefits these aims. The competitive nature of the application process and reports of schemes being over-subscribed further reinforces this. Another important factor for continued involvement in MSFR schemes was the sense that participants were making a meaningful contribution to the local community, a theme previously identified as an important motivator amongst ‘lay’ CFRs [23, 24]. We did not find a significant advantage in response time through utilisation of MSFRs although this has been found in other smaller studies [11].

The impact of positive interprofessional relationships with local ambulance crews was also a commonly reported motivator and reinforcement of such positive relationships has been previously suggested in the literature as a possible tool for lay CFR retention [25]. There were concerns about personal safety but no experiences of traumatic incidents, for example involving children or violence, as has been observed in other studies of medical students working in prehospital settings [26].

### Strengths and limitations

We investigated MSFRs using data from two ambulance services and five medical schools in England. Our findings characterise the activity of MSFRs in detail for the first time. However, our quantitative data corresponds to the period before the COVID-19 pandemic, which may have subsequently affected the activity and experience of MSFRs due to changes in ambulance demand, MSFR dispatch criteria and the use of Personal Protective Equipment (PPE). In addition, we adopted a convenience sampling method for interviews, thus experienced and enthusiastic MSFRs were likely to have been overrepresented in this study. Further studies may help explore more fully the negative aspects of MSFR work, as our sample was likely biased towards those MSFRS that are particularly active. Such future work could achieve this by purposefully approaching MSFRs who have either dropped out of schemes or those who are less active.

### Implications for policy, practice, and research

MSFR schemes provide a unique opportunity for medical students to gain experience of working in the prehospital environment and of independently managing acutely unwell patients. This study has demonstrated a perception amongst MSFRs that their experience provides benefits to their medical education, outcomes of training and future career as well as benefits to patients and the community. These experiences might otherwise be difficult to gain as part of traditional undergraduate medical education. We would argue that MSFR schemes could be more formally incorporated into existing undergraduate medical training in the future; MSFR training could be delivered to all undergraduate students, widening access to this volunteering opportunity. Medical schools could further consider providing students with specifically allocated time in their teaching schedule to allow them to pursue MSFR opportunities. Further research is needed to investigate whether these activities confer demonstrable improvement in educational or clinical performance.

It is also worth considering the many doctors who have been involved in MSFR schemes as medical students and are now qualified: the perceptions of these doctors on the impact of their MSFR work on their career may provide additional insight into the benefits of the MSFR experience.

## Conclusion

Medical students attend thousands of emergency calls annually. In this capacity, they see and treat a group of patients and presentations that are different from their CFR and ambulance colleagues. Medical students feel their involvement in MSFR schemes improves their education and attainment at medical school through improved communication and clinical skills, decision making and team-working. Further research is needed to demonstrate whether this translates into future educational and clinical performance.

## Data Availability

The data that support the findings of this study are available from East Midlands and South Central Ambulance Foundation NHS Trusts but restrictions apply to the availability of these data, which were used under license for the current study, and so are not publicly available. Data are however available from the authors upon reasonable request and with permission from East Midlands and South Central Ambulance Foundation NHS Trusts and the NHS Research Ethics Committee.
