# Correction: Perceptions and experiences of medical student first responders: a mixed methods study

**DOI:** 10.1186/s12909-022-03884-9

**Published:** 2022-11-29

**Authors:** Andrew Orsi, Adam Watson, Nimali Wijegoonewardene, Vanessa Botan, Dylan Lloyd, Nic Dunbar, Zahid Asghar, A. Niroshan Siriwardena

**Affiliations:** 1 Community and Health Research Unit, University of Lincoln, Lincoln, UK; 2 East Midlands School of Anaesthesia, Health Education England, Leeds, UK; 3 Medical Science Division, University of Oxford, Oxford, UK; 4 Directorate of Healthcare Quality and Safety, Ministry of Health, Colombo, Sri Lanka; 5 Medical School, University of Buckingham, Buckingham, UK; 6 South Central Ambulance Service NHS Foundation Trust, Otterbourne, UK

**Correction: BMC Med Educ 22, 721 (2022)**

**https://doi.org/10.1186/s12909-022-03791-z**

Following publication of the original article [1], we have been informed that authors Andrew Orsi and Adam Watson declare joint first authorship.

The authorship has been updated above and the original article has been corrected.
